# Genetic determinants of cerebrospinal fluid metabolites and risk of Guillain-Barré syndrome: A bidirectional two-sample Mendelian randomization study

**DOI:** 10.1097/MD.0000000000040352

**Published:** 2024-11-08

**Authors:** Xiangjia Qi, Liqian Gao, Lifeng Qi

**Affiliations:** a Department of Neurology, Liaocheng People’s Hospital, Liaocheng, China.

**Keywords:** bidirectional, cerebrospinal fluid metabolites, Guillain-Barré syndrome, two-sample Mendelian randomization

## Abstract

This study aims to investigate the potential causal relationship between cerebrospinal fluid (CSF) metabolites and Guillain-Barré syndrome (GBS) using a bidirectional two-sample Mendelian randomization (MR) approach. Publicly available summary data from genome-wide association studies (GWAS) were utilized for comprehensive analysis. The CSF metabolite GWAS summary data were extracted from a GWAS conducted by Panyard et al encompassing 338 CSF metabolites in European participants (n = 291). GWAS summary statistics for GBS were obtained from the FinnGen consortium (n = 215,931) comprising European populations. The primary method for MR analysis was the inverse variance weighted method. Various sensitivity analyses were conducted to assess the heterogeneity and pleiotropy of the findings. In the forward MR analysis, we identified a causal relationship between 15 CSF metabolites, including ribitol levels (odds ratio = 3.833, 95% confidence interval: 1.949–7.540, *P* = 9.87E−05), and the risk of developing GBS. In the reverse MR analysis, we found a causal relationship between GBS and 21 CSF metabolites, including gamma-glutamylphenylalanine levels (odds ratio = 0.934, 95% confidence interval: 0.904–0.966, *P* = 7.10E−05). No evidence of heterogeneity or horizontal pleiotropy was found in the MR analysis. Our findings suggest that the identified CSF metabolites and metabolic pathways can serve as valuable biomarkers for clinical screening and prevention of GBS. They may also be considered as candidate molecules for future research into the underlying mechanisms and for selecting drug targets.

## 1. Introduction

Guillain-Barré syndrome (GBS), an immune-mediated polyradiculoneuropathy, is the leading cause of acute flaccid paralysis worldwide, with approximately 100,000 new cases occurring each year.^[1]^ Most patients experience a sudden onset of neurological symptoms following an infectious illness, which leads to progressive limb weakness lasting up to 4 weeks before reaching a plateau.^[2]^ GBS remains a serious condition, with a mortality rate of approximately 5% and up to 20% of patients unable to walk independently after 1 year.^[3]^

For psychiatric and neurological disorders, the cerebrospinal fluid (CSF) is particularly relevant. Being in direct contact with the brain and spinal cord and separated from the blood by the blood–brain barrier, CSF can more accurately reflect physiological changes occurring in the central nervous system compared to other sample types.^[4]^ In Alzheimer disease, for example, CSF provides some of the most potent biomarkers for disease onset and progression, such as amyloid-beta and phosphorylated tau.^[5]^ Due to the unique nature of CSF samples, there are currently no studies exploring the relationship between CSF metabolites and GBS.

Observational epidemiological studies often encounter biases such as confounding and reverse causation, which limit their ability to establish causal associations. To address these limitations, instrumental variables (IVs) approaches, such as Mendelian randomization (MR), leverage germline genetic variants as proxies for modifiable exposures, thereby strengthening causal inferences in nonexperimental settings.^[6]^ This study aims to use MR to investigate the potential causal relationship between CSF metabolites and the risk of GBS, providing novel perspectives for GBS intervention.

## 2. Materials and methods

### 2.1. Study design

**Figure 1.**
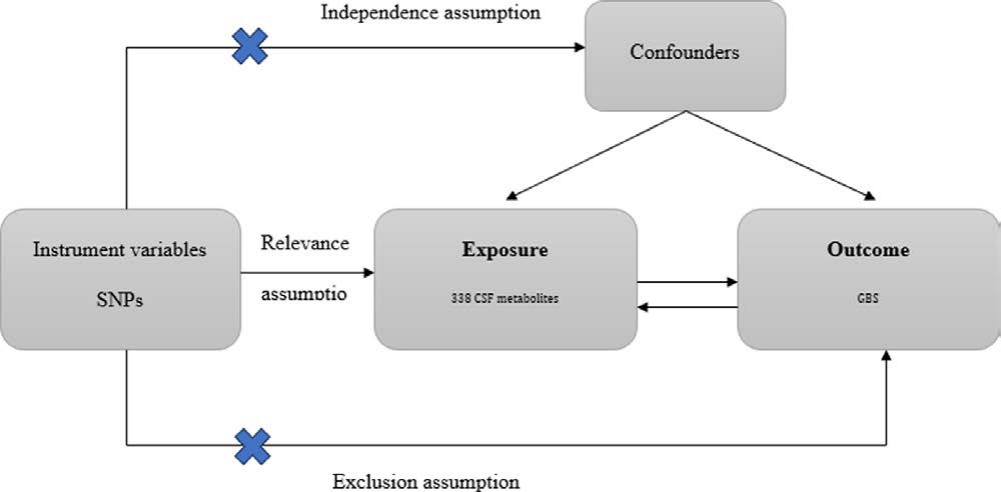
Assumption and design for the MR study. CSF = cerebrospinal fluid, GBS = Guillain-Barré syndrome, MR = Mendelian randomization, SNPs = single nucleotide polymorphisms.

Assumption 1 posits a substantial association between genetic variants and 338 CSF metabolites. Assumption 2 states that these genetic variants are unrelated to confounding factors that influence the exposure-outcome association. Assumption 3 hypothesizes that the genetic variants affect GBS risk solely through their influence on CSF metabolites. Figure 1 presents a brief description of this MR design.

### 2.2. Exposure data

The CSF metabolite genome-wide association studies (GWAS) summary data were obtained from a GWAS study conducted by Panyard et al, which included 338 CSF metabolites measured in 291 European participants. These data were categorized into 8 metabolic groups: amino acids, carbohydrates, cofactors and vitamins, energy, lipids, nucleotides, peptides, and xenobiotics.^[4]^

### 2.3. Outcome data

GWAS summary statistics for GBS were sourced from the FinnGen consortium, focusing on the phenotype “Guillain-Barré Syndrome” (finn-b-G6_GUILBAR). The GWAS included 215,931 adult Finnish subjects, comprising 213 cases and 215,718 controls. As the data were publicly accessible, ethical approval or patient consent was not required for the analysis.

### 2.4. Genetic variants selection criteria

To investigate the causal relationship between CSF metabolites and GBS, IVs were selected using a systematic approach. Initially, single nucleotide polymorphisms (SNPs) that met the genome-wide significance threshold (*P* < 5 × 10^−^⁶) and demonstrated associations with the exposure were identified as potential IVs. A subsequent refinement process, based on linkage disequilibrium parameters such as *r*² and a window size of 10,000 kb (with *r*² < 0.001), was employed to ensure the independence of the selected IVs. Additionally, *F*-statistics were calculated to assess the strength of the IVs, with a threshold set at *F*-statistics >10, which is commonly recommended for MR analysis. This rigorous selection process aimed to reduce bias from weak instrument effects and establish a robust causal inference between CSF metabolites and GBS.

## 3. Statistical analysis

The statistical analyses were carried out using R statistical software (version 4.3.2, http://www.r-project.org/), and MR analyses were conducted using the TwoSampleMR package in R. The results were expressed as odds ratios (OR), reflecting the risk of the outcome per unit change in exposure, along with the corresponding 95% confidence intervals (CI).

For the MR analysis, the *F*-statistic was calculated to measure the strength of each IVs.^[7]^ IVs with *F*-statistics >10 were deemed robust instruments capable of mitigating bias typically associated with weak instruments.^[8]^ The *F*-statistic was calculated using the following formula: *F* = *R*^2^(N − 2)/(1 − *R*^2^), where N = the GWAS sample size for the exposure association, and *R*^2^ = the proportion of variance explained by the genetic variants.

For the primary analysis, we used the inverse variance weighted (IVW) method to estimate the effect of CSF metabolites on GBS, as this method provides precise estimates while accounting for potential heterogeneity among individual variants. However, since the IVW method requires all genetic variants to be valid IVs to ensure accuracy, we supplemented it with MR-Egger and weighted median approaches to evaluate the robustness of the results.^[9,10]^

Heterogeneity was evaluated using Cochran *Q* statistic and its associated *P*-value.^[11]^ Heterogeneity was assessed using Cochran *Q* statistic and its associated *P*-value, and the results were visualized with funnel plots. Cochran *Q* statistic was calculated using the mr_heterogeneity function in the TwoSampleMR package. A *P*-value >0.05 indicated no significant heterogeneity among the genetic IVs for CSF metabolites. Additionally, the leave-one-out method was used to identify and remove any outliers among the SNPs, after which the MR analysis was repeated to strengthen the robustness of the findings.

The MR-Egger intercept analysis was conducted to assess potential directional pleiotropy and to determine whether the intercept was consistent with zero.^[11]^ The MR-PRESSO test was employed to identify outlier variants exhibiting horizontal pleiotropy.^[12]^ The MR-Egger intercept and MR-PRESSO tests were conducted using the mr_pleiotropy and mr_presso functions, respectively, within the TwoSampleMR package in R. A *P*-value >0.05 indicated no evidence of pleiotropy among the genetic IVs for CSF metabolites.

## 4. Results

### 4.1. MR analysis of CSF metabolites on GBS

**Figure 2.**
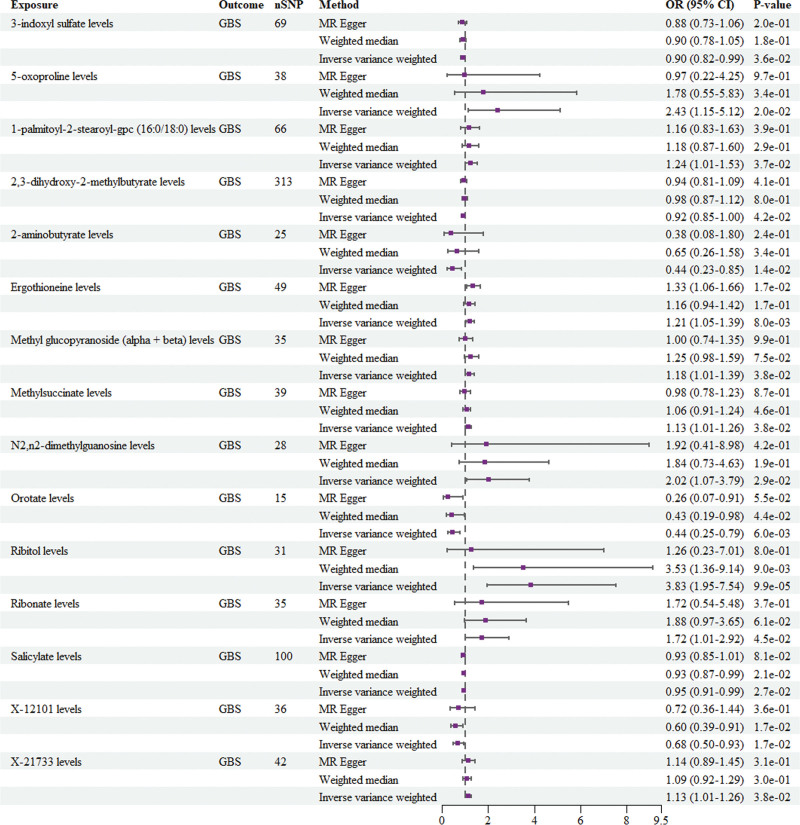
Forest plot for the causal effect of CSF metabolites on the risk of GBS. CI = confidence interval, CSF = cerebrospinal fluid, GBS = Guillain-Barré syndrome, MR = Mendelian randomization, nSNPs = number of single-nucleotide polymorphisms, OR = odds ratio.

**Figure 3.**
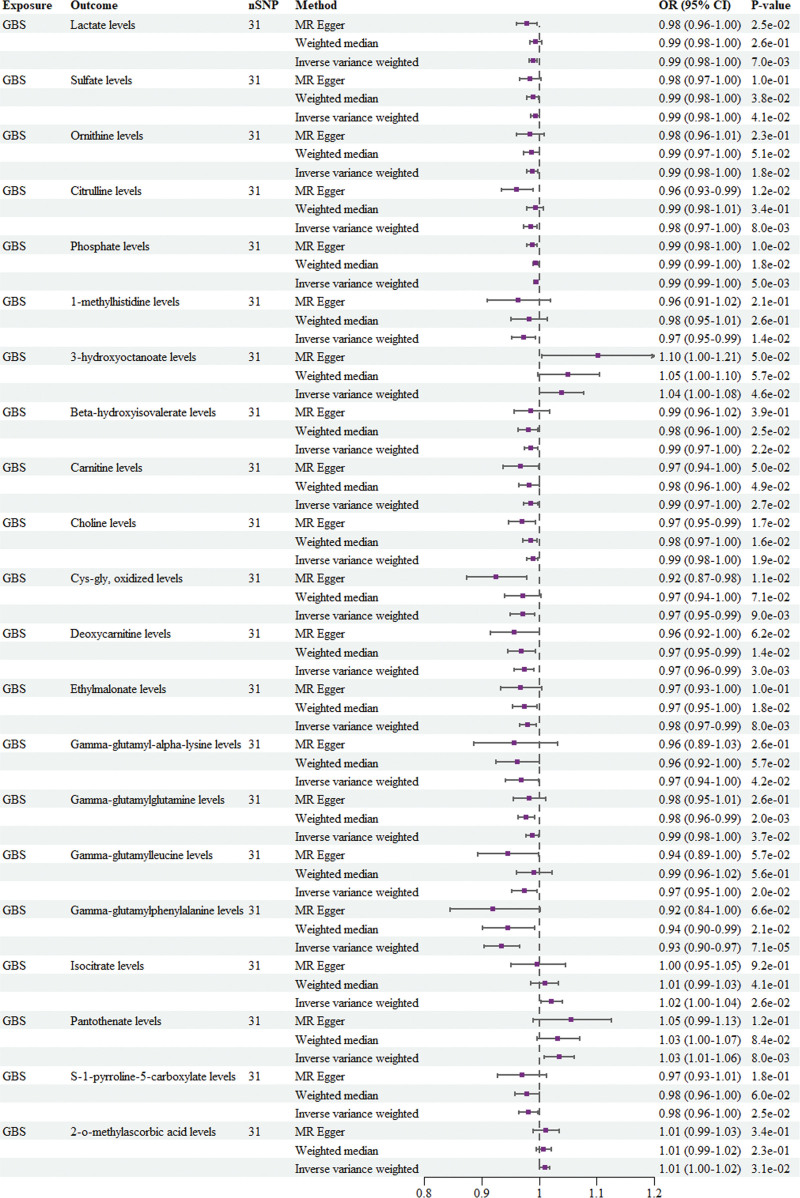
Forest plot for the causal effect of GBS on the CSF metabolites. CI = confidence interval, CSF = cerebrospinal fluid, GBS = Guillain-Barré syndrome, MR = Mendelian randomization, nSNPs = number of single-nucleotide polymorphisms, OR = odds ratio.

This study employed MR analysis to investigate the causal relationships between CSF metabolites and GBS. The findings are shown in Figures 2 and 3. The IVW analysis revealed a statistically significant association between certain CSF metabolites and GBS. Among them, 5-oxoproline levels (OR = 2.426, 95% CI: 1.150–5.120, *P* = .020), 1-palmitoyl-2-stearoyl-gpc (16:0/18:0) levels (OR = 1.244, 95% CI: 1.014–1.527, *P* = .037), ergothioneine levels (OR = 1.208, 95% CI: 1.050–1.390, *P* = .008), methyl glucopyranoside (alpha + beta) levels (OR = 1.184, 95% CI: 1.01–1.389, *P* = .038), methylsuccinate levels (OR = 1.126, 95% CI: 1.007–1.26, *P* = .038), N2, n2-dimethylguanosine levels (OR = 2.017, 95% CI: 1.073–3.791, *P* = .029), ribitol levels (OR = 3.833, 95% CI: 1.949–7.540, *P* = 9.87E−05), ribonate levels (OR = 1.717, 95% CI: 1.011–2.915, *P* = .045), and X-21733 levels (OR = 1.127, 95% CI: 1.007–1.261, *P* = .038) are positively correlated with the risk of developing GBS (Fig. 2). 3-Indoxyl sulfate levels (OR = 0.901, 95% CI: 0.817–0.993, *P* = .036), 2,3-dihydroxy-2-methylbutyrate levels (OR = 0.920, 95% CI: 0.849–0.997, *P* = .042), 2-aminobutyrate levels (OR = 0.441, 95% CI: 0.229–0.849, *P* = .014), orotate levels (OR = 0.445, 95% CI: 0.251–0.789, *P* = .006), salicylate levels (OR = 0.953, 95% CI: 0.914–0.994, *P* = .027), and X-12101 levels (OR = 0.680, 95% CI: 0.4960–0.932, *P* = .017) were negatively correlated with the risk of developing GBS (Fig. 2).

### 4.2. MR analysis of GBS on CSF metabolites

GBS was positively correlated with 3-hydroxyoctanoate levels (OR = 1.039, 95% CI: 1.001–1.078, *P* = .046), isocitrate levels (OR = 1.021, 95% CI: 1.003–1.041, *P* = .026), pantothenate levels (OR = 1.035, 95% CI: 1.009–1.061, *P* = .008), and 2-o-methylascorbic acid levels (OR = 1.01, 95% CI: 1.001–1.019, *P* = .031) (Fig. 3). GBS was negatively correlated with lactate levels (OR = 0.990, 95% CI: 0.983–0.997, *P* = .007), sulfate levels (OR = 0.993, 95% CI: 0.985–1.000, *P* = .041), ornithine levels (OR = 0.988, 95% CI: 0.979–0.998, *P* = .018), citrulline levels (OR = 0.985, 95% CI: 0.973–0.996, *P* = .008), phosphate levels (OR = 0.995, 95% CI: 0.991–0.998, *P* = .005), 1-methylhistidine levels (OR = 0.973, 95% CI: 0.952–0.994, *P* = .014), beta-hydroxyisovalerate levels (OR = 0.986, 95% CI: 0.974–0.998, *P* = .022), carnitine levels (OR = 0.986, 95% CI: 0.973–0.998, *P* = .027), choline levels (OR = 0.989, 95% CI: 0.979–0.998, *P* = .019), Cys-gly, oxidized levels (OR = 0.971, 95% CI: 0.949–0.992, *P* = .009), deoxycarnitine levels (OR = 0.974, 95% CI: 0.957–0.991, *P* = .003), ethylmalonate levels (OR = 0.980, 95% CI: 0.966–0.995, *P* = .008), gamma-glutamyl-alpha-lysine levels (OR = 0.969, 95% CI: 0.941–0.999, *P* = .042), gamma-glutamylglutamine levels (OR = 0.988, 95% CI: 0.977–0.999, *P* = .037), gamma-glutamylleucine levels (OR = 0.974, 95% CI: 0.953–0.996, *P* = .020), gamma-glutamylphenylalanine levels (OR = 0.934, 95% CI: 0.904–0.966, *P* = 7.10E−05), and S-1-pyrroline-5-carboxylate levels (OR = 0.981, 95% CI: 0.965–0.998, *P* = .025) (Fig. 3).

### 4.3. Sensitivity analysis

To comprehensively investigate the correlation between CSF metabolites and GBS, we conducted analyses to assess heterogeneity and pleiotropy (Tables 1 and 2). Since IVW methods are vulnerable to weak IVs bias, a sensitivity analysis showed that the MR-Egger and weighted median estimates were consistent with the directions of the MR estimates. Heterogeneity was assessed using Cochran Q statistic in the MR-Egger regression, and no significant evidence of heterogeneity was found in the effects of the instrumental SNPs. Additionally, the MR-Egger intercept and MR-PRESSO tests did not reveal any horizontal pleiotropy (*P* > .05). Furthermore, leave-one-out analysis and forest plots did not identify any anomalous SNPs (Table 1).

**Table 1:** Heterogeneity and horizontal pleiotropy of the associations between CSF metabolites and the risk of GBS.

Exposure	Outcome	Heterogeneity				MR-PRESSO	Pleiotropy test	
		Inverse variance weighted		MR-Egger		*P* for global test	MR-Egger	
		*Q*	Q_pval	*Q*	Q_pval		Egger_intercept	*P*-value
3-Indoxyl sulfate levels	GBS	63.850	0.620	63.790	0.589	.545	0.009	.808
1-Palmitoyl-2-stearoyl-gpc (16:0/18:0) levels	GBS	57.472	0.735	57.225	0.713	.746	0.017	.620
2,3-Dihydroxy-2-methylbutyrate levels	GBS	299.778	0.680	299.656832874599	0.668	.667	−0.008	.728
2-Aminobutyrate levels	GBS	14.120	0.944	14.081	0.925	.947	0.010	.846
Ergothioneine levels	GBS	28.746	0.988	27.635	0.989	.990	−0.034	.300
Methyl glucopyranoside (alpha + beta) levels	GBS	32.951	0.519	31.212	0.556	.513	0.043	.196
Methylsuccinate levels	GBS	27.135	0.905	25.170	0.930	.9	0.054	.169
Orotate levels	GBS	11.308	0.662	10.404	0.661	.659	0.048	.359
Salicylate levels	GBS	107.277	0.268	106.670	0.258	.273	0.022	.457
X-12101 levels	GBS	42.967	0.167	42.921	0.140	.195	−0.009	.849
X-21733 levels	GBS	34.428	0.756	34.422	0.719	.723	−0.003	.939
N2, n2-dimethylguanosine levels	GBS	27.722	0.425	27.717	0.373	.474	0.003	.944
Ribitol levels	GBS	0.944	0.474	27.927	27.927	.504	0.062	.177
Ribonate levels	GBS	48.097	0.055	0.055	0.0552	.054	2.195	1.000
5-Oxoproline levels	GBS	35.610	35.610	35.610	0.582	.545	0.053	.167

CSF = cerebrospinal fluid, GBS = Guillain-Barré syndrome, MR = Mendelian randomization, *Q* = Cochran Q.

**Table 2:** Heterogeneity and horizontal pleiotropy of the associations between GBS and the CSF metabolites.

Exposure	Outcome	Heterogeneity				MR-PRESSO	Pleiotropy test	
		Inverse variance weighted		MR-Egger		*P* for global test	MR-Egger	
		*Q*	Q_pval	*Q*	Q_pval		Egger_intercept	*P*-value
GBS	Lactate levels	32.418	0.348	30.368	0.396	0.345	0.005	.172
GBS	Sulfate levels	30.713	0.430	29.768	0.426	0.45	0.003	.345
GBS	Ornithine levels	26.522	0.648	26.415	0.603	0.671	0.002	.746
GBS	Citrulline levels	22.093	0.851	18.948	0.923	0.862	0.010	.087
GBS	Phosphate levels	35.078	0.240	31.767	0.330	0.246	0.003	.093
GBS	1-Methylhistidine levels	32.694	0.336	32.546	0.296	0.347	0.004	.719
GBS	3-Hydroxyoctanoate levels	31.856	0.374	29.959	0.416	0.401	−0.025	.186
GBS	Beta-hydroxyisovalerate levels	19.207	0.934	19.206	0.916	0.933	−0.0001	.981
GBS	Carnitine levels	33.388	0.306	31.633	0.336	0.319	0.008	.215
GBS	Choline levels	39.339	0.118	35.689	0.183	0.134	0.008	.096
GBS	Cys-gly, oxidized levels	26.646	0.642	23.346	0.760	0.621	0.020	.080
GBS	Deoxycarnitine levels	27.193	0.613	26.480	0.600	0.654	0.007	.406
GBS	Ethylmalonate levels	27.352	0.604	26.880	0.578	0.648	0.005	.498
GBS	Gamma-glutamyl-alpha-lysine levels	37.453	0.164	37.251	0.140	0.169	0.006	.695
GBS	Gamma-glutamylglutamine levels	36.716	0.186	36.535	0.158	0.214	0.002	.708
GBS	Gamma-glutamylleucine levels	27.985	0.571	26.665	0.590	0.589	0.0127	.260
GBS	Isocitrate levels	37.687	0.158	36.267	0.166	0.155	0.100	.295
GBS	Pantothenate levels	18.627	0.947	18.213	0.940	0.947	−0.008	.525
GBS	S-1-pyrroline-5-carboxylate levels	37.077	0.175	36.669	0.155	0.186	0.005	.574
GBS	2-o-methylascorbic acid levels	23.163	0.808	23.146	0.770	0.799	−0.0006	.895
GBS	Gamma-glutamylphenylalanine levels	19.501	0.929	29	0.912	0.943	0.006	.706

CSF = cerebrospinal fluid, GBS = Guillain-Barré syndrome, MR = Mendelian randomization, Q = Cochran Q.

In the reverse MR analysis, we employed the same methods and found no evidence of heterogeneity or pleiotropy (Table 2), this strengthens the robustness of our conclusions.

## 5. Discussion

Due to the presence of temporal causal relationships and confounding factors, traditional research methods often struggle to fully elucidate the complex relationship between CSF metabolites and GBS. Exploring this relationship through the lens of host genetic variations has emerged as a crucial research avenue. In the forward MR analysis, we identified 15 CSF metabolites that are causally related to the risk of GBS. In the reverse MR analysis, we found a causal relationship between GBS and 21 CSF metabolites. These findings offer new perspectives on the development of GBS and suggest potential avenues for precision medicine in its treatment.

In our MR study, the forward MR analysis indicates a strong causal relationship between Ribitol levels and GBS. Ribitol, a sugar alcohol, plays a critical role in various biological systems, including its involvement in neurological functions and immunological responses. Research indicates that Ribitol has a connection to diseases related to nervous system dysfunction and immune responses, particularly through its role in glycosylation processes, which are crucial for maintaining cell structure and function. In the context of neurological diseases, including those with immune components like GBS, abnormalities in glycosylation can lead to altered nerve cell interactions and immune responses. Specifically, Ribitol is a component of O-mannosyl glycans, which are involved in the proper function of α-dystroglycan, a glycoprotein that plays a role in maintaining the integrity of muscle and nerve cells by connecting them to the extracellular matrix. Deficiencies in glycosylation, particularly in structures like ribitol-5-phosphate in α-dystroglycan, have been linked to congenital muscular dystrophies and other neurodegenerative conditions, highlighting the importance of Ribitol in maintaining nerve cell integrity and preventing immune-mediated nerve damage.^[13,14]^

Furthermore, elevated levels of Ribitol and other polyols in body fluids have been observed in patients with certain metabolic disorders, including those affecting the pentose phosphate pathway, which is essential for proper neuronal function. Although acute neurotoxic effects of Ribitol have not been directly observed, its accumulation due to metabolic defects can lead to broader disruptions in the nervous system, possibly contributing to the development of peripheral neuropathies like GBS. The interplay between Ribitol role in glycosylation and its metabolic dysregulation provides a potential link between its presence and the pathophysiology of neuroimmune conditions.^[15,16]^Given the unique properties of CSF, there are currently no clinical reports linking Ribitol in CSF to the induction of GBS. Further basic research is needed to validate and elucidate the specific pathogenic mechanisms involved.

Gamma-glutamylphenylalanine, a metabolite involved in the gamma-glutamyl cycle, plays a significant role in oxidative stress regulation and has been linked to various immune and neurological conditions. In the context of neuroimmune-related diseases such as GBS, oxidative stress is a critical factor that can exacerbate inflammation and immune responses, which are central to the pathophysiology of GBS.^[3]^ Elevated levels of gamma-glutamylphenylalanine, as observed in studies, have been associated with increased oxidative stress markers and are linked to aging-related diseases, where oxidative damage to nerve cells is a common feature.^[17]^ Research suggests that metabolites like gamma-glutamylphenylalanine, which are products of the glutathione cycle, could contribute to the increased immune response observed in GBS.^[18]^ In this context, oxidative stress may lead to the dysregulation of immune functions, enhancing the autoimmune attack on peripheral nerves, characteristic of GBS. Additionally, gamma-glutamylphenylalanine has been shown to correlate with biological aging markers, suggesting that its dysregulation could play a role in neurodegenerative processes and inflammatory responses. This highlights the potential for gamma-glutamylphenylalanine as a biomarker in both aging-related neuroimmune conditions and autoimmune disorders like GBS.^[19]^

This study has several notable strengths. Firstly, it is the first large-scale MR investigation utilizing random genotype allocation across 2 distinct cohorts, significantly enhancing our ability to reliably identify potential causal relationships between CSF metabolites and GBS and offering new therapeutic possibilities. Secondly, the use of diverse MR analysis techniques effectively reduces the impact of confounding variables, thereby improving the study’s internal validity. Lastly, rigorous primary sensitivity analyses were performed to thoroughly evaluate the assumptions of the MR model, further strengthening the robustness and reliability of our findings.

Our study has several limitations that should be considered. Firstly, we applied a threshold filtering for IVs at a significance level of *P* < 5 × 10^−^⁶, which some might consider relatively lenient. Secondly, our sample consisted exclusively of individuals of European ancestry, limiting the generalizability of our findings to other ethnic or racial groups. Thirdly, GBS is a complex autoimmune disorder with intricate mechanistic foundations. As such, while our MR analysis focused on CSF metabolite levels, these biomarkers may not fully capture disease progression. To gain a more comprehensive understanding, additional basic experiments are needed to validate and elucidate the pathogenic mechanisms involved in GBS.

## 6. Conclusions

In summary, this is the first systematic MR analysis using genome-wide data to evaluate the causal relationship between CSF metabolites and GBS, providing preliminary evidence on the impact of CSF metabolites on GBS risk. Through IVW and multiple sensitivity analyses, a causal relationship between 15 CSF metabolites and GBS risk was confirmed. Additionally, the reverse MR analysis revealed a causal relationship between GBS and 21 CSF metabolites. These findings suggest that these CSF metabolites could serve as useful biomarkers for clinical GBS screening and prevention, as well as potential candidate molecules for future mechanistic studies and drug target selection.

## Acknowledgments

The authors gratefully acknowledge Panyard et al for providing the summary data on CSF metabolites, and extend their appreciation to the FinnGen GWAS database for access to GBS data.

## Author contributions

**Conceptualization:** Lifeng Qi.

**Investigation:** Xiangjia Qi.

**Project administration:** Lifeng Qi, Liqian Gao.

**Software:** Xiangjia Qi.

**Supervision:** Lifeng Qi.

**Validation:** Liqian Gao.

**Writing – original draft:** Xiangjia Qi.
